# Urine Metabolomics Profiling of Lumbar Disc Herniation and its Traditional Chinese Medicine Subtypes in Patients Through Gas Chromatography Coupled With Mass Spectrometry

**DOI:** 10.3389/fmolb.2021.648823

**Published:** 2021-06-09

**Authors:** Letian Shan, Jinying Yang, Shijie Meng, Hongfeng Ruan, Li Zhou, Fusheng Ye, Peijian Tong, Chengliang Wu

**Affiliations:** ^1^ The First Affiliated Hospital, Zhejiang Chinese Medical University, Hangzhou, China; ^2^ Department of Orthopaedics, Zhejiang Xiaoshan Hospital, Hangzhou, China

**Keywords:** metabolomics, lumbar disc herniation, metabolic pathway, GC-MS, Chinese medicine

## Abstract

Lumbar disc herniation (LDH) possesses complex pathogenesis, which has not been well elucidated yet. To date, specific or early diagnosis of LDH remains unavailable, resulting in missed opportunity for effective treatment. According to Traditional Chinese medicine (TCM) theory, LDH can be divided into two subtypes (reality syndrome and deficiency syndrome). The purpose of this study was to analyze the metabolic disorders of LDH and its TCM subtypes and screen out potential biomarkers for LDH diagnosis. Gas chromatography coupled with mass spectrometry (GC-MS) was applied to test the urine samples from 66 participants (30 healthy volunteers, 18 LDH patients with deficiency syndrome and 18 patients with reality syndrome). PCA analysis showed a distinct separation tendency between the healthy subjects and LDH patients but no obvious separation between the different syndromes (reality syndrome and deficiency syndrome) of LDH patients. As a result, 23 metabolites were identified significantly altered in the LDH patients, as compared with the healthy subjects. The altered metabolites belong to amino acid metabolism, nucleic acid metabolism, carbohydrate metabolism, and vitamin metabolism, which are related to osteoporosis and inflammation. Our results indicate metabolic disorders of LDH and thereby propose a group of metabolic biomarkers for potential application in early diagnosis of LDH in clinic, which provide a reasonable explanation for the pathogenesis of LDH.

## Introduction

Lumbar disc herniation (LDH) is a clinically common degenerative spinal disease, which results in deterioration of patients’ life quality and work ability. The pathogenesis of LDH is associated with gender, age, body mass, smoking, heavy loading of spine, and physical activity (Perera et al., 2017). With the change of working and living habits during the development of society, LDH affects an increasing number of young patients (Karademir et al., 2017). The main symptoms of LDH include weakening of muscle strength, lower back pain, radiation pain on one side or in the lower extremities, and sensory disturbances (Pinto et al., 2012; Yang et al., 2015). Many therapeutic approaches, such as non-invasive treatments and surgery, have been developed to treat LDH, relieving pain in patients (Armon et al., 2007; Gibson and Waddell, 2007; Alvi et al., 2018; Choi et al., 2018). The diagnosis plays a key role in the treatment of LDH, which determines the disease progression and affects the treatment efficacy. However, a true gold standard of diagnosis, especially early diagnosis standard, remains unavailable for LDH (Andersson and Deyo, 1996; Jarvik and Deyo, 2002; Lurie et al., 2008). Mostly, imaging diagnosis (CT or MRI) is a mainstay and reliable option, but it works only when patients suffer from LDH at middle- and late-stage (Herzog, 1996; Jarvik and Deyo, 2002). Early stage of LDH occurs with only biochemical or metabolic abnormalities, other than tissue degenerations. Therefore, routine diagnosis is difficult to identify LDH earlier, resulting in missed diagnosis and missed opportunity for early treatment. It suggests a clinical importance and significance of developing a new method for early and more precise diagnosis of LDH.

Traditional Chinese Medicine (TCM) is a complementary and alternative medicine with individualized diagnosis and treatment system (Rioux, 2019). Holistic concept and dialectical treatment are two basic characteristics of TCM, in which syndrome differentiation (*Zheng* in Chinese) profiles an overall body condition of patients (symptoms, feelings, tongue appearance, pulse waves, etc.) and plays an important role in TCM diagnosis and treatment (Bao et al., 2010; Shan et al., 2014). TCM syndromes provide different standard of classification, which may contribute to the early and precise diagnosis of diseases (Jiang et al., 2012). According to the TCM theory, LDH has two main syndromes: 1) reality syndrome with Qi stagnation and blood stasis, wind-cold dampness and stagnation; and 2) deficiency syndrome with liver and kidney deficiency (Nicholson and Lindon, 2008). To fully understand LDH and facilitate its diagnosis, it is better to consider and study the above syndromes with modern medicine.

Previous studies have explored the association of metabolic disorders with intervertebral disc degeneration (IDD), revealing that metabolic syndrome was significantly associated with the development of IDD and accumulation of metabolic syndrome components significantly increased the odds ratio for IDD (Teraguchi et al., 2016; Fujita et al., 2020). Based on the earlier evidences of associations between disc degeneration and metabolism, we put forward a hypothesis that metabonomics can be used to facilitate the understanding of LDH and contribute to the early diagnosis of LDH. Metabonomics is a high-throughput methodology platform for qualitative and quantitative measurement of metabolites in living systems and their dynamic responses to pathophysiological stimuli or genetic modifications (Nicholson et al., 1999). It has been widely used for metabolic profiling of modern diseases and TCM syndromes, which reflects the overall changes of metabolites in line with the holistic concept of TCM (Fernie et al., 2004; Liu et al., 2012). The targets of metabonomics analysis are a variety of low molecular weight metabolites (MW <1000) in blood or urine, serving as the metabolic biomarkers for precise diagnosis of diseases (Lao et al., 2009). Gas chromatography-mass spectrometry (GC-MS) is a predominant technology for metabonomics analysis, with advantages of high sensitivity, peak resolution and reproducibility (Pasikanti et al., 2008). In this study, GC-MS was applied to identify the urine metabolic differences between LDH patients and normal people as well as between two TCM syndromes of LDH patients, aiming at profiling metabolic characteristics and providing potential diagnostic biomarkers of LDH and LDH syndromes. This urine metabonomics might be a new approach for study of LDH, and thus contribute to early diagnosis and specific clinical interventions for LDH patients.

## Materials and Methods

### Chemicals and Instruments

N-methyl-N-(trimethylsilyl) tri-fluoroacetamide (MSTFA), methoxyamine, pyridine and standard compounds were purchased from Sigma-Aldrich (St. Louis, MO, United States). HPLC grade methanol were purchased from Tedia Co., Inc. (Fairfield, OH, United States). Ultrapure water was produced by the Milli-Q ultra-pure water system (Millipore, Billerica, MA, United States).

Agilent 7890/5975C (single quadrupole mass spectrometer) system (Agilent Technologies, United States) was adopted for GC-MS analysis. An Eppendorf 5804 Rcentrifuge (Hamburg, Germany), a CentriVap Concentrator (LABCONCO, Kansas City, MO, United States) and medical syringes (5 ml, Shandong WEGO Group Medical Polymer Co., Ltd., China) were used.

### Subjects

Totally 66 subjects, including 30 healthy volunteers and 36 LDH patients (18 patients with reality syndrome and 18 patients with deficiency syndrome), were enrolled from Zhejiang Provincial Hospital of TCM (The First Affiliated Hospital of Zhejiang Chinese Medical University) with random gender selection (Table 1). Healthy volunteers were selected as the control group with no symptoms, while LDH patients were imagologically diagnosed and selected as the observation group. The clinical parameters of enrolled subjects were monitored at the recruitment phase. The healthy volunteers and LDH patients with concomitant diseases that might affect the outcomes were excluded from this study. The pharmacological treatments on LDH patients were conducted after the sampling step to avoid the interference. The TCM diagnosis was performed by observing physical signs, tongue coating, and pulse profile of each patient, and 18 LDH patients with Qi stagnation and blood stasis were collected as the reality syndrome group (group A), and another 18 LDH patients with liver and kidney deficiency were collected as the deficiency syndrome group (group B). All procedures of this study were approved by the Ethics Committees of the First Affiliated Hospital of Zhejiang Chinese Medical University (Registration Code: 2020-KL-159-02), and the written informed consents were obtained from all subjects.

**TABLE 1:** Basic characteristics of subjects.

Characteristics	Controls (*n*)	LDH patients (*n*)	
		Reality syndrome (A)	Deficiency syndrome (B)
Female	17	10	12
Male	13	8	6
Average age	47 (yrs)	48 (yrs)	49 (yrs)
LDH progress	—	6.3 (yrs)	6.7 (yrs)

### Sample Collection and Preparation

Spot urine samples were collected and stored at −80°C. 50 μl of each sample was added with 75 μl of urease solution (10 mg/ml) and incubated at 37°C for 15 min. After cooling, the mixture was added with 500 μl cold methanol to deposite proteins. The supernatant was collected and dried by CentriVap Concentrator, followed by oximation with 50 μl of methoxamine pyridine solution (20 mg/ml) at 37°C for 90 min and subsequent derivation with 40 μ of MSTFA at 37°C for 60 min. Quality control (QC) sample was an equal mixture of all test samples and was pretreated together with samples.

### GC-MS Analysis

GC-MS metabonomic analysis was conducted on an Agilent 7890/5975C GC-MS system with a 0.25 mm (i.d.) × 30 m × 0.25 μm DB-5MS fused-silica capillary column (5% diphenyl cross-linked 95% dimethylpolysiloxane, Agilent J&W Scientific, CA, United States). The column temperature was initially kept at 70°C for 3 min, and was then increased at a rate of 5°C per min to 300°C for 5 min. The temperature of ion source was set at 200°C. Injection volume of each sample was 1 μl with split ratior of 10:1. Pure helium (99.9996%) was used as the carrier gas with a linear velocity of 40 cm per second. The detector voltage was set at 1.38 kV, and the solvent delay time at 5 min. Full scan mode was employed in the mass range of 33–600 m/z at a rate of 2 spectra per second.

### Data Analysis

Leco Chromatof software (LECO, St. Joseph, MI, United States) was used to process GC-MS data which were converted to CDF format. Peak tables of all samples were merged, and peaks which did not exist in 50% of each group were excluded. Prior to multivariate analyses, total peak area normalization was performed to diminish the influence of uncorrelated factors, and the area of each peak was divided by the total peak area for normalization. MetaboAnalyst (http://www.metaboanalyst.ca/MetaboAnalyst/faces/Home.jsp) was used to perform pattern recognition. Principal component analysis (PCA) with unit variance scaling was used to evaluate the analytical performance, and to obtain an overview of the urine metabolites (Sangster et al., 2006; Gika et al., 2007). Partial least squares-discriminant analysis (PLS-DA) with non-parametric test was conducted to determine the metabolic differences between LDH patients and healthy subjects. Plus, permutation test was used for model validation. The Variable Importance in the Projection (VIP) value was used to determine the potential biomarkers and to estimate the variable contribution (Eriksson et al., 2006). The variables with VIP values beyond 1.5 were considered responsible for the model classification, and the threshold for selecting potential biomarkers was set at *p* < 0.05 and VIP >1.5 (Gu et al., 2012). The metabolite identification was performed by comparing the mass spectra of metabolites with NIST mass spectral database. Some standard compounds were purchased for the conformation of metabolite structure. MetaboAnalyst was applied for the pathway analyses (Xia et al., 2009; Xia et al., 2012).

## Results

### Metabolic Profiling and Multivariate Statistical Analysis

**FIGURE 1:**
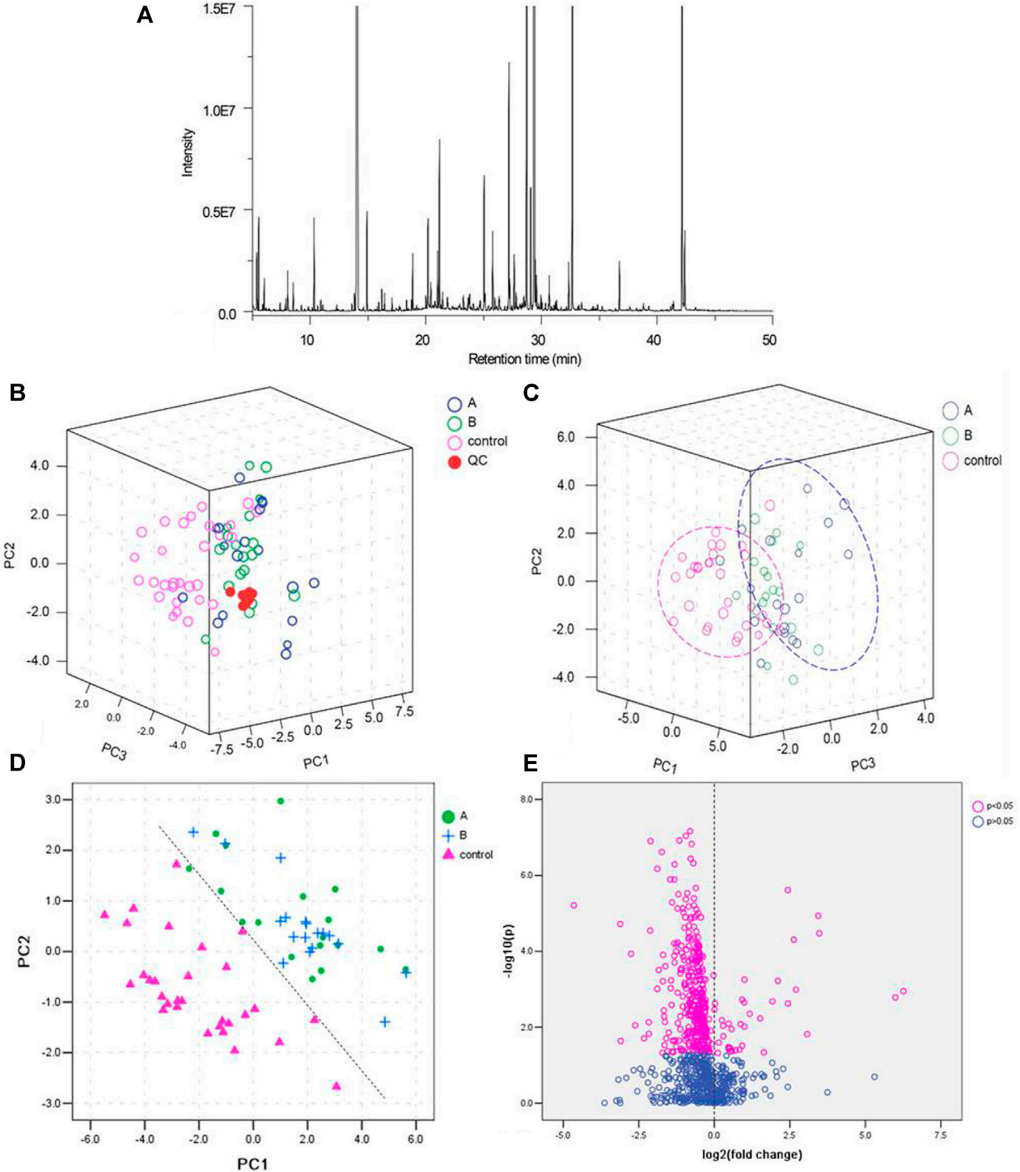
Quality control (QC) of GC-MS analysis and partial least squares discriminant analysis (PLS-DA) of the urine samples. **(A)**: Total ion chromatograms (TICs) of the urine samples; **(B)**: PCA score scatter plots of QC samples from healthy subjects (control) and LDH patients (A with reality syndrome; B with deficiency syndrome); **(C)**: PCA score scatter plots of urine samples from healthy subjects (control) and LDH patients (A with reality syndrome; B with deficiency syndrome); **(D)**: PLS-DA score scatter plot of urine samples from healthy subjects (control) and LDH patients (A with reality syndrome; B with deficiency syndrome); and **(E)**: volcano plot of urine metabolites in healthy subjects and LDH patients.

Representative total ion chromatograms (TICs) showed 855 metabolic peaks in the urine samples (Figure 1A). To validate the GC-MS, QC samples was tested and their PCA score plots tended to cluster together, indicating accuracy and reliability of methodology (Figure 1B). Further PCA analysis showed a distinct separation tendency between the healthy subjects and LDH patients but no obvious separation between the different syndromes (reality syndrome and deficiency syndrome) of LDH patients (Figure 1C). For PCA analysis, PC1, PC2, and PC3 explained 25.2, 9.1 and 4.8% of the total variances, respectively. Considering the little difference between the TCM syndromes of LDH patients, subsequent analyses were conducted only for the comparison between the healthy subjects and LDH patients. To determine the metabolic variations associated with LDH, a PLS-DA model was constructed for multivariate analysis of urine samples between the healthy subjects and LDH patients (Supplementary Figure S1A). PC1 and PC2 totally explained more than 80% of the total variance (Supplementary Figure S1B). As shown in Figure 1D, the PLS-DA score plot indicated a distinct separation between the healthy subjects and LDH patients, while the separation between the different syndromes was not obvious. As shown in Figure 1E, the result of non-parametric test indicated that 81 of 855 metabolites were significantly differed between the healthy subjects and LDH patients (*p* < 0.05). However, considering the little difference between the TCM syndromes of LDH patients, subsequent analyses were conducted only for the comparison between the healthy subjects and LDH patients.

**FIGURE 2:**
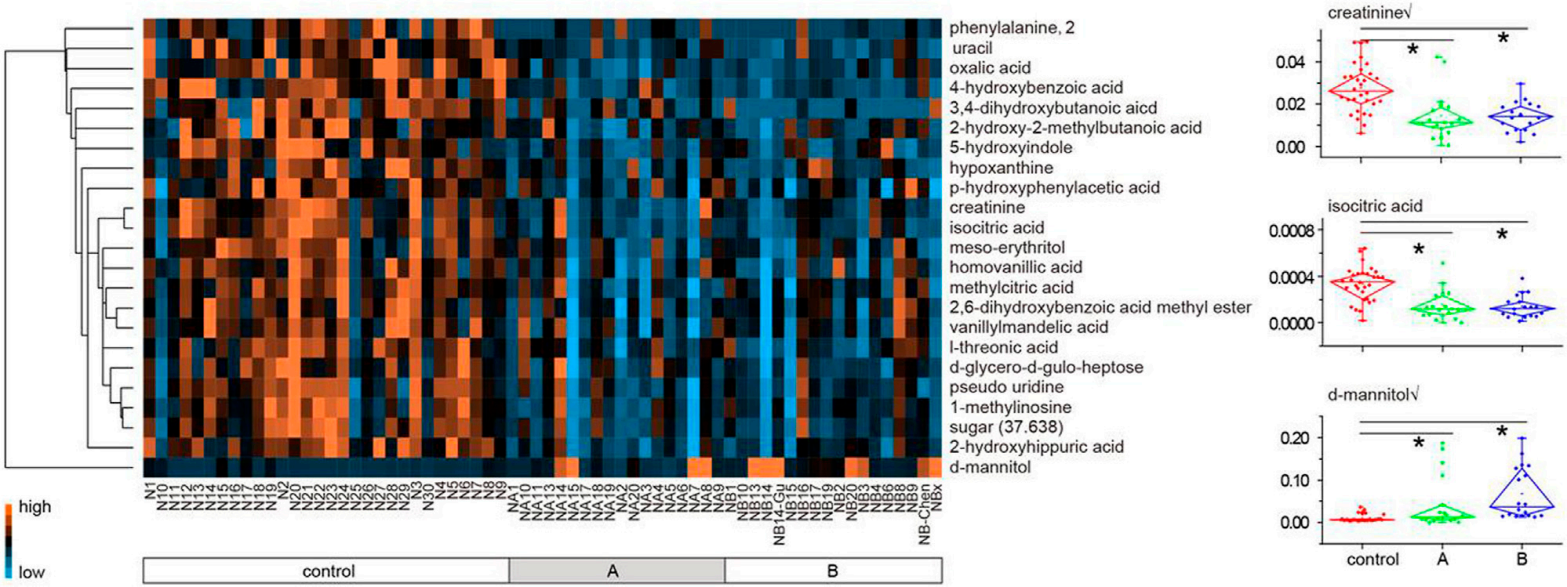
Heat map of 23 metabolites differed between the healthy subjects and LDH patients (A plus B). Each metabolite is represented by a single row of colored boxes, and columns represent different samples. Metabolic levels are represented by different colors. The red label shows high levels in urine, while the green labels show low intensity relative to the median metabolic levels.

On basis of the VIP threshold (VIP >1.5, Supplementary Figure S1C) and non-parametric test (*p* < 0.05), 23 metabolites were identified with significant difference between the healthy subjects and LDH patients (Table 2). A heat map was generated to visualize the altered pattern of the significantly differed metabolites (Figure 2). Most of the metabolites (22 of 23) were down-regulated in the urine of LDH patients, including uracil, oxalic acid, 4-hydroxybenzoic acid, 3,4-dihydroxybutanoic acid, 2-hydroxy-2-methylbutanoic acid, 5-hydroxyindole, hypoxanthine, p-hydroxyphenylacetic acid, creatinine, isocitric acid, meso-erythritol, homovanillic acid, methylcitric acid, 2,6-dihydroxybenzoic acid methyl ester, vanillylmandelic acid, l-threonic acid, pseudo uridine, 1-methylinosine, sugar (37.638), phenylalanine, d-glycero-d-gulo-heptose, and 2-hydroxyhippuric acid, while d-mannitol was up-regulated. None of the metabolites were found significantly differed between the TCM syndromes of LDH patients.

**TABLE 2:** Characterization of the significantly different metabolites between the healthy subjects and LDH patients.

Compounds	RT	VIP	Fold change (patients/control)	Main metabolic pathway
1-methylinosine	46.201	1.702	0.587^a,d^	Unknown
2,6-dihydroxybenzoic acid methyl ester	27.459	1.512	0.737^a,d^	Unknown
2-hydroxy-2-methylbutanoic acid	10.09	1.677	0.652^a,d^	Unknown
2-hydroxyhippuric acid	31.964	2.204	0.520^a,c^	Unknown
3,4-dihydroxybutanoic acid	18.321	2.702	0.331^b^	Unknown
4-hydroxybenzoic acid	22.986	2.626	0.448^a,c^	Phenylalanine metabolism
5-hydroxyindole	24.691	2.149	0.491^a,c^	Tryptophan metabolism
Creatinine√	21.177	2.131	0.521^a,c^	Arginine and proline metabolism
D-glycero-D-gulo-heptose	37.389	1.595	0.690^a,c^	Unknown
d-mannitol√	29.345	1.789	5.404^a,c^	Fructose and mannose metabolism
Homovanillic acid	26.142	1.666	0.700^a,c^	Tyrosine metabolism
Hypoxanthine√	26.825	1.647	0.524^a,c^	Purine metabolism
Isocitric acid	27.218	2.324	0.430^a,c^	Tricarboxylic acid (TCA) cycle
l-threonic acid^b^	21.022	1.563	0.710^a,c^	Unknown
Meso-erythritol√	20.168	1.939	0.553^a,c^	Unknown
Methylcitric acid	27.483	1.686	0.667^a,c^	Unknown
Oxalic acid√	10.164	2.416	0.574^a,c^	Glyoxylate and dicarboxylate metabolism
Phenylalanine	22.88	1.575	0.223^b^	Phenylalanine metabolism
p-hydroxyphenylacetic acid	23.253	1.691	0.631^b^	Phenylalanine metabolism
Pseudo uridine^b^	36.746	1.757	0.596^a,c^	Pyrimidine metabolism
Sugar (37.638)	37.638	1.83	0.559^a,c^	Starch and sucrose metabolism
Uracil√	15.787	1.679	0.415^a,c^	Pyrimidine metabolism
Vanillylmandelic acid	28.385	1.616	0.694^a,c^	Unknown

^a^ *p* < 0.01, ^b^ *p* < 0.05, ^c^adjusted *p* < 0.01 and ^d^adjusted *p* < 0.05.

√means the structure of metabolite has been validated by standard compound.

### Pathway Analysis

**FIGURE 3:**
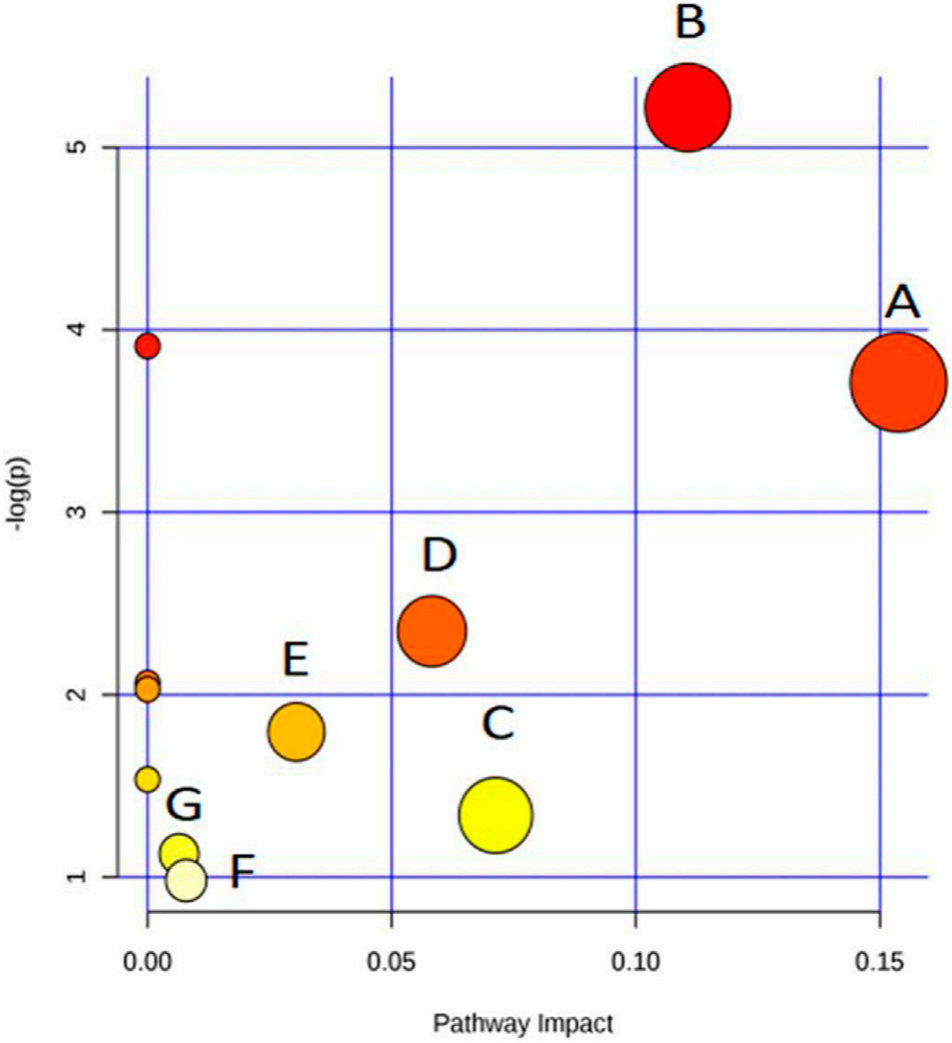
Overview of pathway analysis **(A)** glyoxylate and dicarboxylate metabolism, **(B)** tyrosine metabolism, **(C)** pyrimidine metabolism, **(D)** tricarboxylic acid (TCA) cycle, **(E)** ubiquinone and other terpenoid-quinone biosynthesis, **(F)** purine metabolism, and **(G)** arginine and proline metabolism.

**FIGURE 4:**
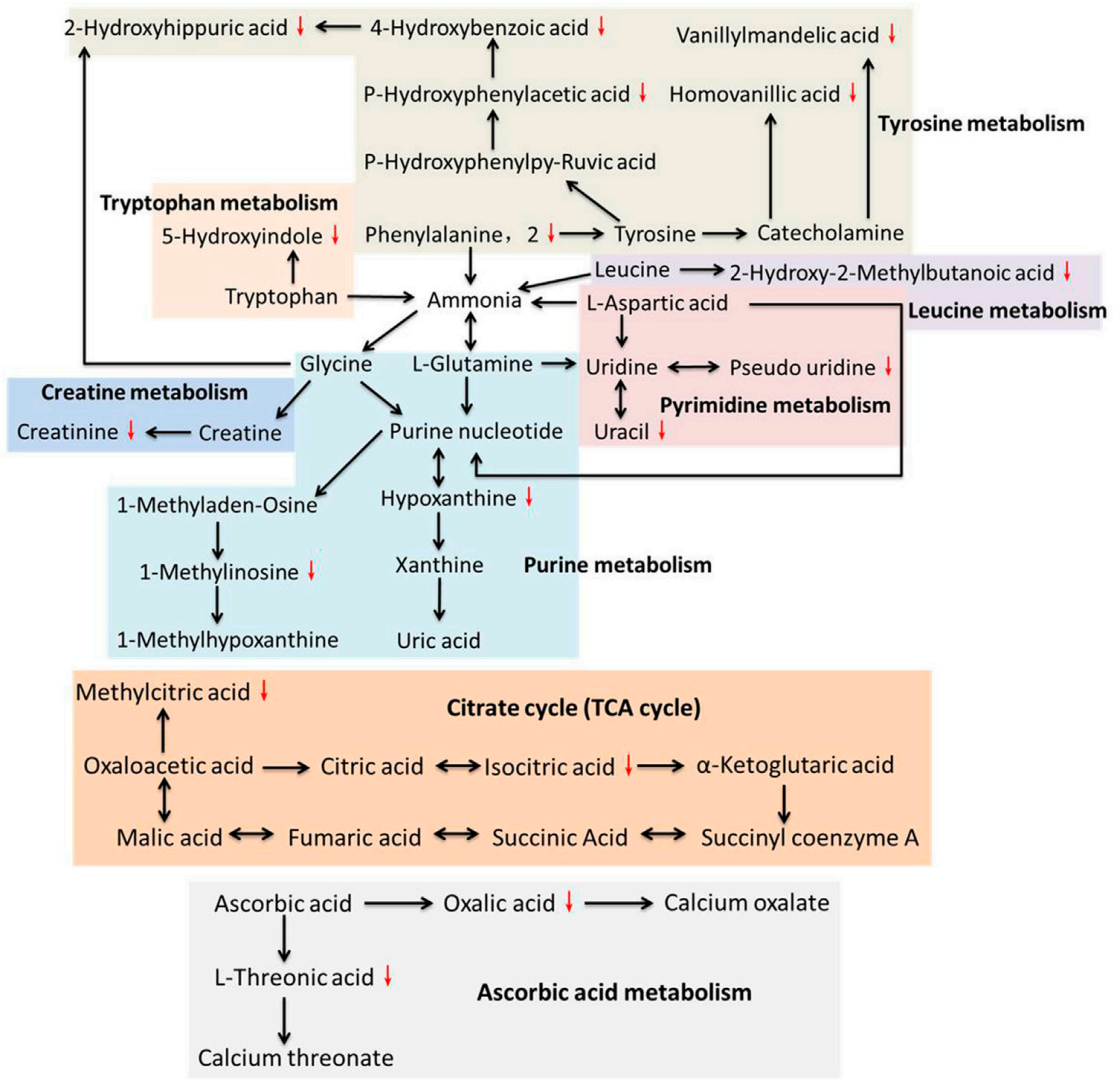
Relationships among the principal metabolic pathways altered in the urine of LDH patients.

Figure 3 was generated by the tool MetaboAnalyst 2.0 and contains all matched metabolic pathways associated with the identified metabolites. It was constructed by *p* values on the Y-axis (from pathway enrichment analysis) and pathway impact values on the X-axis (from pathway topology analysis). Pathway enrichment analysis was conducted, relating to quantitative enrichment analysis based on the concentration levels, using GlobalTest and GlobalAncova methods, in which *p* values were approximated according to the asymptotic distribution. Pathway topology analysis was conducted to estimate the importance of biological pathways among the overall pathway structure using two well-established node centrality measures (betweenness centrality and degree centrality). The importance of each metabolite node was measured with regard to the total pathway importance. In Figure 3, the node color was based on its *p* value, and the node size was determined based on its pathway impact value. Accordingly, the main metabolic pathways in LDH patients were glyoxylate and dicarboxylate metabolism, tyrosine metabolism, pyrimidine metabolism, TCA cycle, ubiquinone and other terpenoid-quinone biosynthesis, purine metabolism, and arginine and proline metabolism. The deductive pathways and metabolic connections for the identified metabolites were structured in Figure 4.

## Discussion

Low back pain is a common cause of morbidity, with approximately 80% of the population experiencing backache during their lifetime (Momenzadeh et al., 2019). LDH is one of the main causes of low back pain, which is nowadays increasing in the younger population due to the obesity and inappropriate posturing (Manchikanti et al., 2014; Chen et al., 2019). Previously, modern medicine believed that mechanical compression was the only cause of LDH pain (Huang et al., 2019). Recently, increasing evidences suggest that the pathophysiology of LDH is attributed not only to the pressure on the nerve roots, but also to a complex interplay of inflammatory, immunological, and metabolic processes (Stafford et al., 2007). For instance, high levels of phospholipase A2 was found in herniated nuclear material of patients with radicular pain, provoking an intense inflammatory reaction (Saal et al., 1990; Franson et al., 1992). Clinical studies showed high levels of IL-6, IL-8, and prostaglandin E2 in discs of LDH patients with low back pain (Burke et al., 2002). Moreover, TNF-α has been found to play prominent role in the pathophysiological events of LDH, leading to nerve dysfunction and pain when nucleus pulposus is approximated to lumbar nerve roots (Stafford et al., 2007). A recent review has analyzed in detail the modulatory role of inflammation in LDH, notably placing inflammation as a good prognostic indicator of spontaneous regression of LDH (Cunha et al., 2018). Therefore, the pathogenesis of LDH is, to some extent, biochemical. In our previous study, we profiled the plasma metabolic alterations in LDH patients, by using GC-MS based metabolomics (Shan et al., 2014). The altered plasma metabolic pathways in LDH patients were mainly amino acid metabolism (arginine and proline metabolism, cysteine and methioninemetabolism, glycine, serine and threoninemetabolism), carbohydrate metabolism (starch and sucrose metabolism, fructose and mannose metabolism, galactose metabolism, etc.), nitrogen metabolism, and lipid metabolism, mostly associated with death of nucleated cells, breakdown of proteoglycan and collagen, inflammation, and pain (Shan et al., 2014). To complement the urine metabolic profile of LDH, this study conducted GC-MS based metabolomic comparison between the healthy subjects and LDH patients and between different TCM syndromes. We identified 23 differential metabolites from the urine of LDH patients, which were involved in various biological pathways such as amino acid metabolism (arginine and proline metabolism, tyrosine metabolism, and ubiquinone and other terpenoid-quinone biosynthesis), carbohydrate metabolism (TCA cycle, glyoxylate and dicarboxylate metabolism), and nucleotide metabolism (pyrimidine metabolism and purine metabolism). It can be seen that most of the altered metabolites are directly or indirectly related to each other, of which tyrosine, glycine, purine nucleotide and uridine, as node molecules, have more connections with other metabolites in pathways of amino acid metabolism, while ascorbic acid and oxaloacetic acid are node molecules in pathways of carbohydrate metabolism (Figure 4). Among those pathways, glyoxylate and dicarboxylate metabolism with an impact factor of 0.15 and tyrosine metabolism with an impact factor of 0.11 were found to be the most relevant pathways to LDH. The results suggested the modulatory role of biochemical metabolism in LDH, in which the mentioned metabolites and pathways might determine the metabolic disorders associated with LDH.

It is known that aggrecan, link protein and core protein, containing large amounts of acidic amino acids, constitute cartilage and serve to maintain disc anatomy and functions (Neame and Barry, 1994; Vilim and Fosang, 1994; Melrose et al., 1997). Of these, link protein is composed of high levels of phenylalanine and tyrosine which activate a large spectrum of physiological functions in the bone system (Neame et al., 1985). LDH generally occurs with chronic inflammation and osteophyte hyperplasia, in which phenylalanine metabolism plays an important role (Neame et al., 1985). Phenylalanine acts as an important participator in the process of bone metabolism, which correlated negatively with bone mass and positively with bone resorption activity (Roth et al., 2019). It has been found to be associated with osteoporosis (Malkawi et al., 2018). In this study, our data indicates a significant reduction of urine phenylalanine and its downstream metabolites, such as homovanillic acid, vanillylmandelic acid, p-hydroxyphenylacetic acid, 4-hydroxybenzoic acid, and 2-hydroxyhippuric acid, suggesting an association between osteoporosis and LDH. A proton magnetic resonance spectroscopy study of human disc herniation has determined that decreased level of creatinine might be related to intervertebral disc pathology (Garseth et al., 2002), the result of which was consistent with our data that creatinine was significantly decreased in LDH patients. In addition, low level of creatinine was also associated with low bone mineral density (Yamada et al., 2017; Komorita et al., 2018). As metabolites of nucleic acid, hypoxanthine, 1-methylinosine, uracil, and pseudo uridine were also found significantly reduced in the LDH patients, suggesting the disorder of nucleic acid metabolism participating in the pathological changes of LDH. To date, little study has reported the relationship between nucleic acid metabolism and LDH. In addition to amino acid metabolism and nucleic acid metabolism, carbohydrate metabolism was also found significantly altered in the LDH patients. Of those metabolites, isocitric acid and methylcitric acid are essential substances in the TCA cycle, and their reduction with LDH may indicate an increased aerobic metabolism with inflammatory processes in disc pathology (Garseth et al., 2002). Moreover, oxalic acid and l-threonic acid, the metabolites of vitamin metabolism, were found to be reduced in the urine of LDH patients. Some studies have reported the reduction of l-threonic acid with low bone mineral density, although no study has shown the relationship between vitamin metabolism and LDH (He et al., 2005; Sun et al., 2016). The above findings suggested that the osteoporosis and inflammation-related alterations of amino acid metabolism, nucleic acid metabolism, carbohydrate metabolism, and vitamin metabolism might be associated with the occurrence or development of LDH, and the identified metabolites might serve as potential metabolic biomarkers for the diagnosis of LDH.

The other metabolites, such as 2-hydroxy-2-methylbutanoic acid, 3,4-dihydroxybutanoic aicd, 2,6-dihydroxybenzoic acid methyl ester, 5-hydroxyindole, d-glycero-d-gulo-heptose, meso-erythritol, and d-mannitol, were also found to be significantly altered with LDH. However, no evidence supports their relevance to LDH or lumbar back pain, which requires further investigations to elucidate their mechanism of action in LDH. Since the above biomarkers are not specific for LDH, it may be better to group them as a ‘‘biomarker combination’’ for their application in the clinic. In sum, the present study is supportive to facilitate the understanding of pathogenesis of LDH and the screening of metabolic biomarkers for early diagnosis of this disease. Moreover, urine sample has advantage over blood sample as source of biomarkers, due to its non-invasive sampling, and combining urine biomarkers with blood biomarkers would be a promising way to improve the accuracy and precision of diagnosis.

## Conclusion

This study applied GC-MS in combination with multivariate statistical analysis for investigating the metabolic profile in LDH and its TCM subtypes. We analyzed urine samples from 66 eligible participants (30 healthy volunteers, 18 LDH patients with deficiency syndrome and 18 LDH patients with reality syndrome). As a result, 81 metabolites were found significantly altered in the LDH samples, 23 of which were identified. Most of them were reduced in the LDH samples. Nevertheless, few differences were found between the LDH reality syndrome and LDH deficiency syndrome, suggesting the inadequacy of urine metabonomics approach for separation of different TCM subtypes of LDH. By combining with our previous study, we confirmed that metabolomics method is insufficient to distinguish TCM types of LDH, but our findings revealed the metabolic disorders of LDH and thereby propose a group of metabolic biomarkers for potential application in early diagnosis of LDH in clinic. The altered metabolites belong to amino acid metabolism, nucleic acid metabolism, carbohydrate metabolism, and vitamin metabolism, which are related to osteoporosis and inflammation. These findings provide a reasonable explanation for the pathogenesis of LDH. Further studies are needed to clarify the underlying mechanism of these metabolic processes in the development of LDH.

## Data Availability

The original contributions presented in the study are included in the article/Supplementary Material, further inquiries can be directed to the corresponding author.
