# Supplementary material for: Urine Metabolomics Profiling of Lumbar Disc Herniation and its Traditional Chinese Medicine Subtypes in Patients Through Gas Chromatography Coupled With Mass Spectrometry
Source: Front Mol Biosci. 2021 Jun 9;8:648823. doi: 10.3389/fmolb.2021.648823 (PMC8220151; doi:10.3389/fmolb.2021.648823)
Supplement: Supplementary file 1 [file Table1.DOCX]

Table S1. GC-MS detection information and *P* values for identified metabolites

| Peak id | RT (min) | Identification | *P* value | FDR-adjusted *P* value |
| --- | --- | --- | --- | --- |
| 83 | 10.164 | oxalic acid | < 0.001 | < 0.001 |
| 686 | 31.964 | 2-hydroxyhippuric acid | < 0.001 | < 0.001 |
| 393 | 22.986 | 4-hydroxybenzoic acid | < 0.001 | < 0.001 |
| 609 | 29.345 | d-mannitol | < 0.001 | < 0.001 |
| 338 | 21.177 | creatinine | < 0.001 | < 0.001 |
| 538 | 27.218 | isocitric acid | < 0.001 | < 0.001 |
| 453 | 24.691 | 5-hydroxyindole | < 0.001 | 0.001 |
| 830 | 37.638 | sugar (37.638) | < 0.001 | 0.002 |
| 808 | 36.746 | pseudo uridine | < 0.001 | 0.002 |
| 189 | 15.787 | uracil | < 0.001 | 0.003 |
| 332 | 21.022 | l-threonic acid | < 0.001 | 0.003 |
| 503 | 26.142 | Homovanillic acid | < 0.001 | 0.003 |
| 824 | 37.389 | D-Glycero-D-gulo-Heptose | < 0.001 | 0.004 |
| 306 | 20.168 | meso-erythritol | < 0.001 | 0.004 |
| 528 | 26.825 | hypoxanthine | < 0.001 | 0.005 |
| 578 | 28.385 | vanillylmandelic acid | < 0.001 | 0.005 |
| 546 | 27.483 | methylcitric acid | < 0.001 | 0.006 |
| 82 | 10.090 | 2-hydroxy-2-methylbutanoic acid | 0.001 | 0.010 |
| 545 | 27.459 | 2,6-dihydroxybenzoic acid methyl ester | 0.001 | 0.018 |
| 957 | 46.201 | 1-methylinosine | 0.002 | 0.025 |
| 406 | 23.253 | p-hydroxyphenylacetic acid | 0.012 | 0.141 |
| 389 | 22.880 | phenylalanine | 0.015 | 0.170 |
| 254 | 18.321 | 3,4-dihydroxybutanoic aicd | 0.020 | 0.218 |
