# Supplementary figures and images for: Urine Metabolomics Profiling of Lumbar Disc Herniation and its Traditional Chinese Medicine Subtypes in Patients Through Gas Chromatography Coupled With Mass Spectrometry
Source: Front Mol Biosci. 2021 Jun 9;8:648823. doi: 10.3389/fmolb.2021.648823 (PMC8220151; doi:10.3389/fmolb.2021.648823)

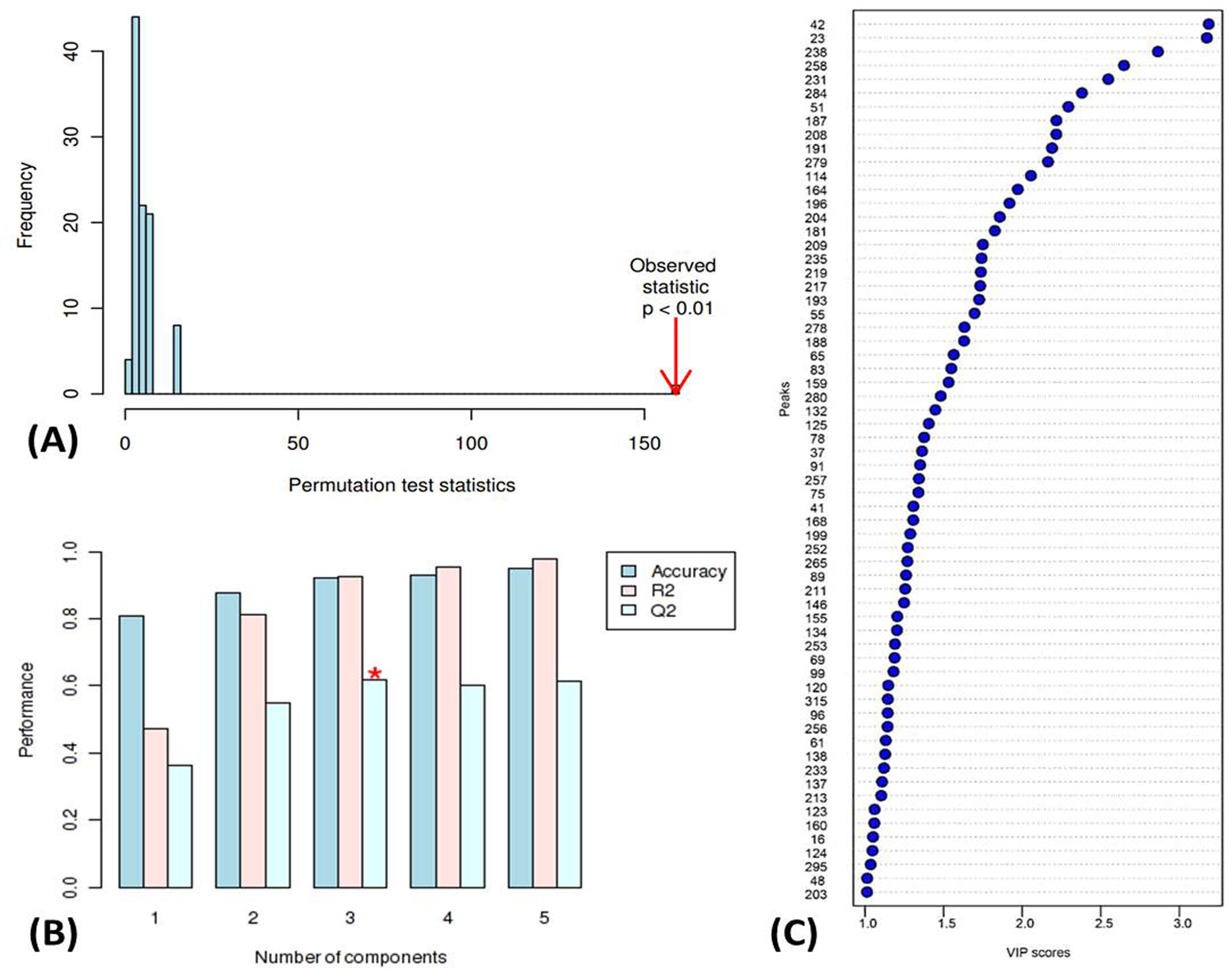

Supplement: Supplementary file 2 [file Image1.JPEG]
